# A Scoping Review of Risk Factors for Thunderstorm Asthma in Pediatric Patients

**DOI:** 10.7759/cureus.115107

**Published:** 2026-08-24

**Authors:** Suraya Foster, Nora Antova, Adam Usher

**Affiliations:** 1 Respiratory Medicine, Gloucestershire Hospitals NHS Foundation Trust, Gloucester, GBR; 2 Respiratory Medicine, University of Bologna, Bologna, ITA

**Keywords:** climate change, pediatrics, public health, risk factors, thunderstorm asthma

## Abstract

Thunderstorm weather conditions are known to trigger asthma-like symptoms in susceptible patients, causing spikes in the use of paramedical, primary care, and hospital services. While risk factors for thunderstorm asthma (TSA) in adults have been studied in both original research and systematic reviews, there has been less focus on the risk profile of pediatric patients. With epidemic TSA (ETSA) events predicted to increase in frequency and severity as climate change progresses, it is more crucial than ever to identify those children most at risk so that appropriate public health education, parent and family education, and conservative management can be initiated. This scoping review followed the PRISMA-ScR checklist. We searched Embase, Ovid MEDLINE, and Scopus for primary research that assessed risk factors or strong predictive associations for TSA in patient cohorts that included children. A total of 39 studies met our inclusion criteria; of these, just five focused exclusively on pediatric populations. We found that ETSA events may trigger a first-time asthma presentation in a large proportion of pediatric patients, especially those with atopic conditions such as allergic rhinitis or eczema. In some studies that included both children and adults, younger age groups were among those most affected by TSA; however, analysis was not routinely age-stratified. These findings highlight a critical gap in the literature, limiting the ability to define a pediatric-specific risk profile. Further research is needed to inform targeted prevention strategies, clinical management, and public health preparedness.

## Introduction and background

Thunderstorm asthma (TSA) refers to an acute asthma exacerbation triggered by thunderstorm weather conditions. The unique environmental conditions that accompany thunderstorm events favor the breakup of pollen into paucimicronic starch granules by osmotic shock, which are subsequently concentrated by wind gusts. As a result, large amounts of allergen-carrying particles are inhaled and lodge deep in the lower airways, triggering bronchospasm [1]. Epidemic TSA (ETSA) is defined as multiple presentations of acute asthma attacks or bronchospasms immediately after a thunderstorm [2]. The most severe ETSA event reported to date occurred in Melbourne in 2016, resulting in over 3,500 excess hospital presentations for respiratory complaints and 10 deaths [3]. Australia is particularly represented in the TSA literature due to the high prevalence of asthma, high levels of grass pollen, and climatic conditions that favor the occurrence of thunderstorms during peak pollen seasons [4]. Although a disproportionate number of ETSA events occur in Australia, they have also been reported in the Middle East [5], China [6], Europe [7], and the USA [8]. With climate change driving an increase in both extreme weather events and the seasonal index of airborne pollen, ETSA events are predicted to continue increasing in frequency and severity [9].

Two recent reviews have explored trends in TSA across the globe, focusing, among other things, on risk factors for presentation to emergency services, hospital admission, and death [9,10]. In their 2021 review, Price et al. found that patients of Asian ethnicity, those with a history of allergic rhinitis, and those with allergen-specific IgE were more susceptible to TSA [10]. Established use of inhaled corticosteroids was a protective factor. While many individuals experiencing TSA did not have a prior diagnosis of asthma, those patients with diagnosed asthma, particularly those with poorly controlled asthma, were at higher risk of severe symptoms requiring hospital admission.

Thunderstorm conditions are known to result in higher numbers of pediatric acute asthma presentations, but, to our knowledge, no reviews have focused exclusively on assessing risk factors for presenting with TSA in this patient population [11]. Children represent a distinct population in the context of thunderstorm-associated asthma. For one, age-related differences in airway physiology and immune development influence how asthma manifests in pediatric patients [12]. Children’s aeroallergen exposure may differ also, as they may spend large amounts of time outdoors during peak pollen seasons. Additionally, some pediatric patients may lack a prior asthma diagnosis and may not be prescribed regular preventive therapy, thus increasing vulnerability to exacerbations [13]. Finally, access to care in pediatric populations is commonly mediated by caregivers, which can influence the timing of presentation, highlighting the importance of identifying specific risk factors for pediatric populations and sharing these findings through parent and patient education [14].

The purpose of this scoping review is to explore what is known about patient-related and environmental factors associated with TSA in pediatric patients. The aim is to identify those most at risk, potentially informing clinical management, patient and parent education, and public health preparedness.

## Review

Methods

The research question for this scoping review was developed using the Population, Concept, Context (PCC) framework [15]. The population of interest comprised children as well as mixed-age cohorts in which pediatric patients were included. The concept of interest was TSA, and the context included ETSA events across all geographical and healthcare settings. The focus of this review was therefore to synthesize what is currently known about TSA and its risk factors among pediatric patients.

The decision to answer this question in the form of a scoping review was motivated by multiple factors. The majority of the literature on TSA focuses on adult patients; even when TSA studies include children in their cohorts, analysis of risk factors is not typically age-stratified. Pediatrics-only studies are few in number, making a systematic review unfeasible. Additionally, while the studies included in this paper all focus on TSA, they differ slightly in exposures and outcomes, for example, analyzing either pollen or fungal spores as an exposure or ED admissions or general practitioner (GP) contacts as an outcome. Therefore, a scoping review was selected as the most appropriate methodology to map out what is currently known about pediatric TSA within a heterogeneous body of literature.

A scoping review was carried out using the PRISMA-ScR checklist [16,17]. No protocol was registered a priori for this review. Furthermore, no formal critical appraisal or statistical analysis was performed, as this was a scoping review. Several search term configurations were tested in an attempt to capture all relevant literature on the topic. The final search string was “thunderstorm*” or “storm*” within three words of “asthma” or “wheeze” (using the Boolean operator ADJ3), executed in April 2026. Embase, Ovid MEDLINE, and Scopus electronic databases were used for the primary search. Backward citation searching was not completed because this was a resource-constrained scoping review. Non-English-language papers were excluded for practical feasibility. There was no restriction on publication date due to the infrequent nature of TSA events. Gray literature was excluded due to feasibility constraints.

Initial title and abstract screening was performed by two reviewers and was blinded. Full-text review of the remaining papers was then carried out by two reviewers according to set inclusion/exclusion criteria. Disagreements were resolved by a third party; this process was also blinded. The data charting process involved the PRISMA-ScR diagram and checklist. Data extracted from each study included a brief description of the study, the age group captured by the study, and any patient-specific and environmental risk factors (or strong associations) mentioned in the study’s conclusion. These data were captured in a summary table that was completed and checked by both reviewers. The data charting process was tracked via the PRISMA-ScR diagram. The PRISMA-ScR checklist was used as a guiding structure for the selection process.

To be included in our final selection of evidence sources, the studies were required to meet the following criteria: (1) be peer-reviewed primary research; (2) explore patient and/or environmental risk factors for TSA; and (3) include pediatric patients. Conversely, studies were excluded if they were not in English, were not primary research, or did not include pediatric patients in a meaningful way. Nonclinical studies, such as those involving animals, were also excluded. While there was no strict age cutoff for assessing which studies did or did not include children, these authors took the view that studies that only assessed patients aged 14, 15, or 16 years and older did not meaningfully assess pediatric patients within their cohorts, not least because many health services begin transitioning patients to adult care at these ages. Cohorts with age cutoffs of 14, 15, or 16 years were thus excluded from this review. A summary of the studies in this category is provided in Appendix A. The final number of studies included in our review was 39.

Results

Study Selection

**Figure 1:**
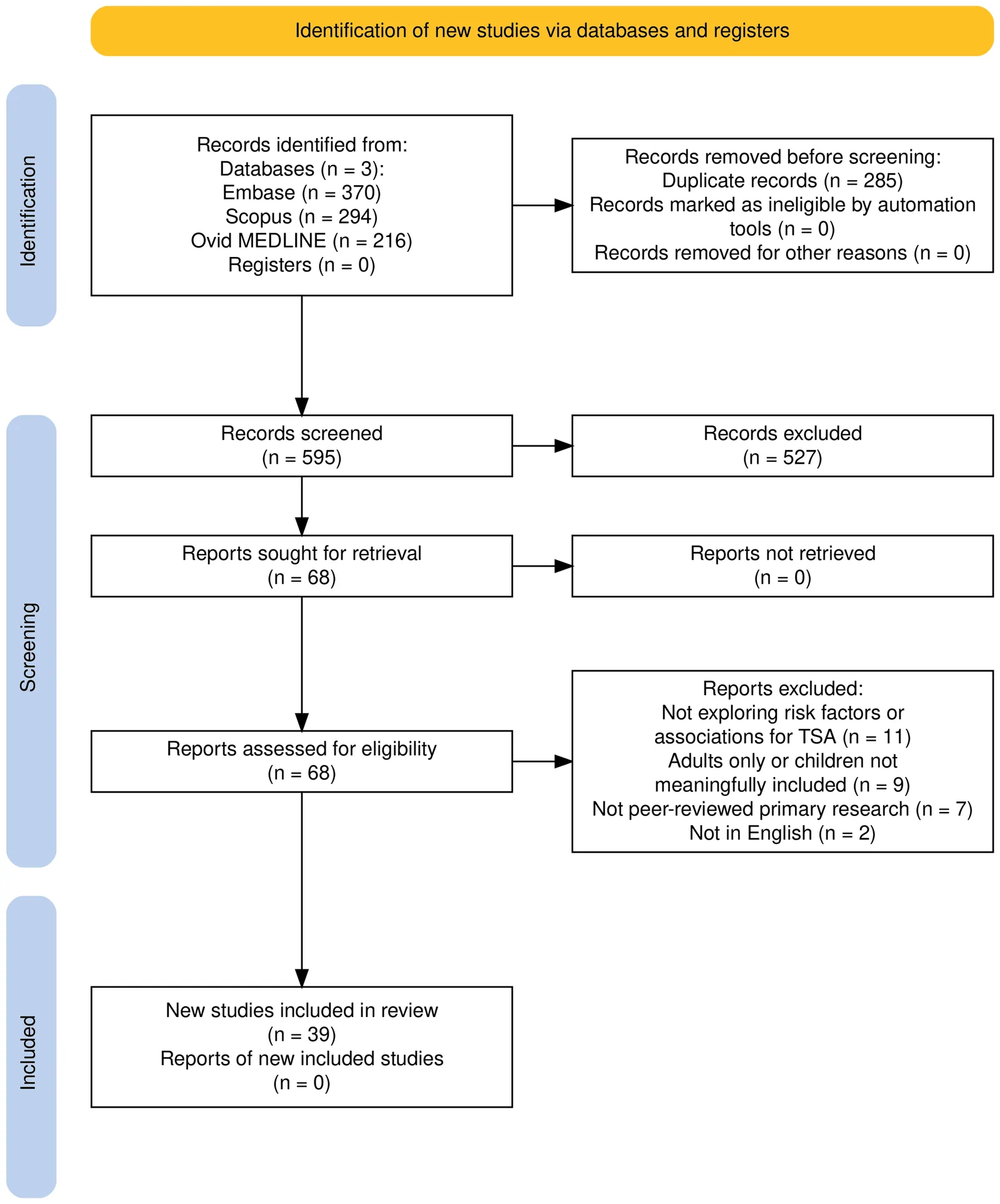
PRISMA flow diagram of final study selection.

The database search yielded 880 records, of which 595 remained after removal of 285 duplicate records, as illustrated by the PRISMA flow diagram (Figure 1). Of these, 527 records were excluded during screening, and 68 full-text articles were assessed for eligibility by two reviewers. Disagreements were resolved by a third reviewer where required. Following full-text assessment, 39 studies were included in the review. The majority of the selected studies comprised mixed adult and pediatric populations (n = 34), with a small number focusing exclusively on pediatric cohorts (n = 5). This highlights the limited number of pediatric-specific studies in the literature. The 39 papers were tabulated, and the following information was extracted: age range of the patient cohort (“Pediatric,” “Mixed Cohort,” or “Mixed Cohort; Age-Stratified”), a summary of study methodology, and key findings. The findings of each included study were taken from the Results and Conclusion sections of each paper and separated into “Environmental Factors” and/or “Patient-Specific Factors” associated with TSA. 

The heterogeneity of the included studies, particularly with regard to methodology, meant that not all studies explicitly determined risk factors for TSA. Many studies, lacking control or comparison groups in their design, found only strong associations with the development of TSA, rather than risk factors. Both types of study were included in this scoping review due to the paucity of literature available on TSA; however, this allows our review to draw overall conclusions about associated factors only, not risk factors.

Mixed-Cohort Studies

The 34 mixed-cohort studies were primarily retrospective and observational, analyzing ED or primary care presentations, as well as syndromic surveillance and environmental data (Table 1). Several survey-based studies were also included. A variety of geographical regions were represented, including Australia (n = 11), the UK (n = 9), China (n = 4), North America (n = 4), Iran (n = 3), and other locations (n = 3). Australia commands disproportionate focus in TSA studies because it was the location of the 2016 Melbourne ETSA event, a devastating crisis that resulted in over 3,500 ED attendances [3].

**Table 1:** Mixed-cohort studies assessing risk factors or associations for TSA. ETSA, epidemic thunderstorm asthma; GP, general practitioner; TSA, thunderstorm asthma

Reference	Overview of study design	Age range	Associated environmental factors	Associated patient-specific factors
Thien et al. (2018) [3]	Retrospective analysis of environmental and patient data for the Melbourne, Australia, ETSA event	Mixed cohort	High grass pollen levels; thunderstorm weather events	Prior asthma diagnosis; Asian or Indian ethnicity; grass pollen sensitization; allergic rhinitis; low inhaled corticosteroid therapy use
Lyne et al. (2025) [4]	Retrospective time-series study of asthma ambulance call-outs, ED presentations, and hospital admissions in South Australia between 2003 and 2017	Mixed cohort; age-stratified	Not found: high pollen levels	-
Forouzan et al. (2014) [5]	Survey study of 2,000 patients who presented with acute bronchospasm to the ED in Iran after a 2013 ETSA event	Mixed cohort; age-stratified	-	Young adults
Liu et al. (2026) [6]	Retrospective single-center study of inpatients during a 2022 ETSA event in Yulin, China (n = 93)	Mixed cohort	Thunderstorm weather event immediately after a high-pollen day; high mugwort pollen levels	Allergic rhinitis; mugwort sensitization; no prior asthma diagnosis; moderately severe asthma
Park et al. (2022) [8]	Time-series study of 63,789 asthma-related ED visits across 64 parishes in Louisiana, USA, from 2010 to 2012	Mixed cohort; age-stratified	Thunderstorm weather events; higher precipitation and lower temperatures	Children
Hao et al. (2026) [18]	Retrospective outbreak study analyzing inpatient cohorts during two ETSA events in Yulin, China, in 2022 (n = 93) and 2025 (n = 28)	Mixed cohort	Thunderstorm weather event; high mugwort pollen levels	Allergic rhinitis; mugwort sensitization; undiagnosed asthma or airway hyperresponsiveness
Hughes et al. (2026) [19]	National syndromic surveillance study of >300,000 asthma-type NHS contacts in England in May and July 2023	Mixed cohort	Thunderstorm weather events; high grass pollen levels; hot weather	Allergic rhinitis
Lang et al. (2025) [20]	Retrospective environmental/geographical analysis of ETSA-affected Chinese cities (Hohhot, Baotou, Yinchuan, and Yulin)	Mixed cohort	Plateau/desert geography; high Artemisia pollen levels; sunny, dry conditions preceding the thunderstorm event	Artemisia sensitization
Lin et al. (2024) [21]	Retrospective environmental study of 10.7 million respiratory ED visits, including 2.3 million asthma visits, in New York State from 2005 to 2018	Mixed cohort; age-stratified	Thunderstorm weather events; power outages; high grass, ragweed, or tree pollen levels; high relative humidity; high levels of particulate matter with an aerodynamic diameter ≤2.5 μm (PM2.5) and ozone; extreme temperatures	Patients with more comorbidities; uninsured/self-paid patients and rural residents; school-age children, older adults, and Hispanic patients
Shoushtari et al. (2024) [22]	Cross-sectional study in Ahvaz, Iran, of 284 TSA subjects and 267 non-TSA symptomatic controls	Mixed cohort; age-stratified	Thunderstorm weather events	Allergic rhinitis; no previous asthma history; bronchial hyperresponsiveness and positive methacholine challenge
Fan et al. (2024) [23]	Retrospective case series of 155 TSA patients in Hohhot, China, after a 2023 thunderstorm event	Mixed cohort	High Artemisia pollen levels	Allergic rhinitis; Artemisia sensitization; no or suboptimal preventer inhaler treatment
Simunovic et al. (2023) [24]	Longitudinal observational study of 20,900 asthma ED presentations in Brisbane, Australia, from 2016 to 2020	Mixed cohort; age-stratified	High grass pollen levels	Female patients
Price et al. (2023) [25]	Environmental analysis of asthma ED presentations from five hospitals in Melbourne during the 2016 ETSA event	Mixed cohort	High pollen levels; high humidity; temperature drops; possible ozone role	-
Lõhmus et al. (2022) [26]	Time-series study of 737,377 respiratory outpatient visits in Stockholm County, Sweden, from 2001 to 2017	Mixed cohort	High birch pollen levels	-
Smith et al. (2022) [27]	Retrospective observational study of ETSA events in the Minneapolis-St. Paul metropolitan area over 11 years	Mixed cohort	Lightning; high pollen levels	-
Elliot et al. (2021) [28]	Retrospective observational study of a single ETSA event in England using syndromic surveillance data	Mixed cohort	Thunderstorm weather events; high grass pollen levels	-
Foo et al. (2020) [29]	Three-year single-center questionnaire-based longitudinal study in Melbourne, Australia	Mixed cohort	-	Low adherence to inhaled corticosteroid therapy or asthma action plan
Bannister et al. (2020) [30]	Case-control study of 81 case hospital days and 157 hospital-day controls in Melbourne, Australia	Mixed cohort	Convergence lines	-
Rad et al. (2019) [31]	Environmental sampling and TSA referral data during periods of thunderstorms in Ahvaz, Iran	Mixed cohort	High pollen levels; high air pollution levels	-
Hew et al. (2019) [32]	Retrospective study of patients presenting to the ED of major Melbourne health services during the 2016 Australian ETSA event	Mixed cohort	-	History of asthma; recent admission for asthma; Asian patients born locally; protective: Asian patients born overseas
Darvall et al. (2018) [33]	Retrospective multicenter observational study of patients admitted to intensive care during the Victoria, Australia, 2016 ETSA event	Mixed cohort	-	Young; history of asthma
Venables et al. (1997) [34]	Analysis of pollen, meteorological, GP, and A&E data during an ETSA event in the London area	Mixed cohort	-	Specific IgE to grass pollen
Hajat et al. (1997) [35]	Analysis of data from 45 GP practices, pollution and pollen data, and meteorological data in the London area over three years	Mixed cohort; age-stratified	Not found: grass pollen levels; not found: air pollution	-
Higham et al. (1997) [36]	Retrospective surveillance study of GP deputizing service calls during a June 1994 ETSA event in England, Wales, and Scotland	Mixed cohort	Thunderstorm exposure	Allergic rhinitis; prior asthma diagnosis
Newson et al. (1997) [37]	Analysis of asthma admissions, meteorological records, and pollen data in England over four years	Mixed cohort	High grass pollen counts; very large sferic (lightning flash) densities	-
Khademi et al. (2026) [38]	Retrospective comparative study of three ETSA events in Victoria, Australia	Mixed cohort	-	21-40 years old; immunological and allergic conditions
Smith et al. (2024) [39]	Retrospective analysis of sex- and age-associated risk of TSA (n = 142,333) over 11 years in the Minneapolis-St. Paul, MN, USA, area	Mixed cohort	High pollen levels	Males 18-44 years old; females 45 years old and older
Okyere-Mensah et al. (2024) [40]	Matched case-control study (41 cases and 82 controls) conducted in two health facilities in Ghana	Mixed cohort	-	History of allergic conditions; history of asthma
Davidson et al. (1996) [41]	Retrospective study of 640 patients presenting to 12 EDs in the London area during an ETSA event	Mixed cohort	High grass pollen levels	Young adults; history of atopy; undiagnosed asthma; not on inhaled corticosteroid therapy
Anderson et al. (2001) [42]	Retrospective analysis of geographical data and asthma admissions during 32 thunderstorm weather events in Wales, UK	Mixed cohort	High ozone levels; high temperatures; not found: grass pollen levels, spore levels, or rainfall	-
Girgis et al. (2000) [43]	Retrospective case-control study (n = 183) of patients attending Wagga Hospital in Australia for asthma during thunderstorms	Mixed cohort	-	History of allergic rhinitis; allergy to rye grass pollen; not taking inhaled corticosteroids if diagnosed with asthma
Losappio et al. (2011) [44]	Study of 20 patients who presented to the ED during an ETSA event in Italy in 2010	Mixed cohort	-	Olea europaea sensitization
Silver et al. (2018) [45]	Time-series ecological study based on asthma admissions to Melbourne metropolitan hospitals over a 16-year period	Mixed cohort	High grass pollen levels	-
Packe and Ayres (1985) [46]	Retrospective study of the hospital and primary care records of 106 patients and the meteorological records associated with an ETSA event in Birmingham, England, in 1983	Mixed cohort	High fungal spore (*Didymella exitialis *and *Sporobolomyces*) levels; not found: air pollution levels	-

Assessment of risk factors in studies of TSA focused on environmental factors (n = 12), patient-specific factors (n = 9), or both (n = 13). Environmental factors were highly location-dependent, with most studies exploring the link between TSA and endemic pollen or spore levels. High levels of grass pollen were the most commonly reported predictive factor for TSA events in the context of thunderstorms. Weather trends were variable; while some studies found warm temperatures [19,42] and high humidity [8,25] to increase TSA risk, others reported that cooler temperatures [8] and dry environments [20] increased the risk of TSA. Finally, air pollution was explored in several studies; however, there was no consensus as to whether high pollution levels increased the risk of developing a TSA event [21,31,35,46].

Among patient-specific factors, results conflicted as to which age groups were most at risk for TSA; while some studies showed older adults were more likely to present with TSA [21], other studies found children or younger people to be at higher risk [8,21,33,41], while others still found no preferential effect on children [39]. Some studies assessed subsets of patient cohorts, such as those admitted to intensive care or those diagnosed with life-threatening asthma, finding that patients with doctor-diagnosed, poorly controlled asthma were at highest risk for critical illness [33]. Interestingly, although an existing asthma diagnosis was regularly reported to confer a higher risk for severe TSA, thunderstorm conditions have also been demonstrated to “unmask” undiagnosed asthma, with many patients presenting for the first time [6,18,22,41]. Finally, several studies highlighted a past medical history of allergic rhinitis or other atopic illness as a risk factor for presenting with TSA across age groups [3,6,18,19,22,23,36,40,41,43], with some going so far as to identify specific IgE to common aeroallergens as patient-specific risk factors [34,44].

Notably, although children were typically included in mixed-cohort studies, age-stratified analyses were not often performed (n = 7/34), limiting the ability to draw pediatric-specific conclusions. The studies that did categorize patients by age often limited analysis to the distribution of those affected by TSA, which, as outlined above, yielded conflicting results [33,38,39]. The few studies that explored age-stratified risk factors suggested that school-age children and the elderly may be most affected by the conditions that promote ETSA events [8,21]. In contrast, the small number of pediatric-focused studies provided more detailed insights into clinical presentation, risk factors, and outcomes in children.

Pediatric-Specific Studies

In our literature search, just five studies assessed risk factors for TSA in pediatric cohorts. Three studies focused on clinical risk factors, while two explored environmental associations.

In two of the pediatric cohort studies, affected children were most commonly school-aged, with a predominance of male patients. In a study conducted in London, UK, 75% of cases were male, with a median age of eight years (range, 2-15 years) [47], and in a study conducted in Yulin, China, the majority of the analyzed cohort were also male, with a median age of seven years (range, 3-14 years) [48].

In both studies, the severity of presentations most commonly ranged from moderate to severe, with occasional life-threatening exacerbations. In the UK cohort, 59% of cases were classified as severe, although there were no deaths or need for endotracheal intubation [47]. In contrast, the Chinese study showed a predominance of moderate asthma exacerbations, but a substantial number of admissions following the TSA event [48]. Despite the acute severity of symptoms, no pediatric deaths were reported across these studies.

In both clinically focused studies, over half of the children affected by TSA had no prior diagnosis of asthma or history of wheeze. This suggests that thunderstorm weather events may “unmask” previously undiagnosed asthma. In the pediatric cohort from Yulin, China, 76% of the first-time wheeze presentations occurred in children with a history of allergic rhinitis [48]. The UK cohort did not analyze the first-time wheeze group separately and instead found that 44% of all children presenting with wheeze had a prior history of eczema or allergic rhinitis [47]. This difference may be attributed to a variety of factors, including slightly different study designs, ETSA event variability, and different geographic locations; the Yulin study looked at hospitalized children, whereas the UK study included all TSA presentations, irrespective of hospitalization.

A different study conducted in Beijing, China, provides a more mechanistic perspective on pediatric TSA. When compared with a group of age- and sex-matched patients with well-controlled asthma, TSA patients were found to have higher levels of CD62L^low^ eosinophils, which were found to be already primed to produce higher levels of inflammatory cytokines. The study also found a higher rate of polysensitization and grass pollen sensitization among children with TSA [49]. These findings should be interpreted with caution, however, as there was no healthy comparison group in the study design, making it unclear whether the findings truly reflect a TSA-specific phenotype or simply a broader acute exacerbation phenotype.

Additionally, there were two studies that analyzed environmental risk factors. In an Ontario, Canada, study analyzing environmental risk factors, a stronger association was found between TSA and increased levels of fungal spores, whereas no such association was observed with air pollutants [50]. In contrast, a different environmental study conducted in Yulin, China, found a stronger association between higher levels of pollen and pediatric outpatient visits for asthma, wheeze, and rhinitis, particularly during the autumn pollen season [51]. All five pediatric studies are detailed in Table 2.

**Table 2:** Studies exploring pediatric risk factors for TSA. TSA, thunderstorm asthma

Reference	Overview of study design	Associated environmental factors	Associated patient-specific factors
Stewart et al. (2024) [47]	Retrospective observational study of 68 presentations with TSA to the ED in London, United Kingdom	-	57% had no prior asthma diagnosis; 44% had a history of atopy
Xu et al. (2021) [48]	Retrospective observational study of 51 patients hospitalized with TSA among 391 total presentations to hospital during a TSA event in 2018 in Yulin, China	-	94% of hospitalized patients had positive IgE against mugwort pollen; 67% of hospitalized patients had a history of allergic rhinitis; 76% of hospitalized patients presented with moderate asthma; males were more commonly affected
Miao et al. (2026) [49]	Case-control immunological study of 16 children with TSA and 26 children with well-controlled asthma	-	Polysensitization and grass pollen sensitization; history of allergic rhinitis; higher levels of CD62L^low^eosinophils
Dales et al. (2003) [50]	Retrospective time-series analysis of approximately 4,000 patients/year presenting with asthma symptoms at Ontario Children’s Hospital between 1993 and 1997	Thunderstorm weather conditions; high fungal spore and pollen levels	-
Deng et al. (2026) [51]	Environmental time-series analysis of 21,224 pediatric outpatient visits for allergic rhinitis, asthma, or wheeze from 2020 to 2022 in Yulin City, China	High pollen levels; lightning; daytime with a lag of zero to two days after thunderstorm weather conditions	-

Discussion

Summary of Evidence

Thirty-nine studies were included in our final review of what is known about pediatric risk factors for TSA. Many of the 34 mixed-cohort studies highlighted the importance of high local pollen and spore levels in predicting a rise in patients presenting with asthmatic symptoms during thunderstorms [3,6,18-21,23-28,31,37,39,41]. An existing diagnosis of asthma [3,32,33,36,40], poorly controlled asthma symptoms or suboptimal treatment [3,23,29,32,41,43], and a past medical history of allergic rhinitis or other atopic conditions [3,6,18,19,22,23,36,38,40,41,43] were consistently reported as patient-specific risk factors for both the development and severity of TSA.

These patient-specific factors were reflected to an extent in the five pediatric studies from China and the UK. First, the paucity and variable nature of environmental studies in pediatric cohorts make it difficult to draw any broader conclusions about environmental risk factors for TSA in children across multiple regions or climates [50,51]. As with the mixed cohorts, a disproportionately high number of pediatric patients affected by TSA did not have a prior asthma diagnosis, although these undiagnosed patients were less likely to experience severe or life-threatening asthma [48]. A strong atopic history, such as allergic rhinitis and eczema, was a predictor of TSA [47-49].

Impact of Climate Change

ETSA events are likely to increase with climate change [9]. One mechanism is the prolongation of pollen seasons, with spring starting increasingly early and ending later, and winters becoming warmer. It is also likely that pollen levels will significantly increase in the coming decade [52], potentially increasing vulnerability in patients with established risk factors. One of the major associated factors for TSA according to this study is elevated pollen concentrations. Pollen types vary by geographical area and climate and may play an asymmetrical role in ETSA events depending on location; for example, Anderson et al. (2001) found no correlation between ETSA events in Wales and grass pollen levels [42].

Another mechanism driving ETSA events is thunderstorm weather conditions themselves. A study by Romps et al. predicted that lightning strikes are likely to increase by approximately 12% for every 1°C increase in temperature [53]. Moreover, according to the IPCC, human-induced global warming is at an all-time high, with one forecasting model estimating a projected temperature increase of 1.56 °C by the year 2050 [53,54]. As warmer air holds moisture well, and moisture is a necessary ingredient for thunderstorms, this warming effect will also drive an increase in thunderstorm weather [55].

Due to the expected increase in pollen levels and thunderstorms in the coming years, further research is crucial to assess the clinical and environmental risk factors for the development of TSA. Specific attention should be paid to regional factors, such as pollen types and seasonal weather variation.

Research Context

Asthma management is experiencing a paradigm shift away from broad population advice and toward individualized patient treatment plans [56,57]. This move toward precision medicine aims to target treatments based on individual patient circumstances, including environment and biomarkers, thus improving health outcomes. The Thunderstorm Asthma in Seasonal Allergic Rhinitis (TAISAR) study by Douglass et al. (2022) was a multicenter study in Australia that explored risk factors for TSA and presentation to hospital in a cohort of adults with self-reported seasonal allergic rhinitis (SAR) [58]. The TAISAR study found that 35% (n = 228) of patients with SAR had experienced symptoms of TSA. The researchers demonstrated that high ryegrass-pollen-specific IgE concentration, eosinophilia, and high fractional exhaled nitric oxide level were all risk factors for the development of TSA in patients with SAR, whether those patients presented to hospital or not. These findings focus the profile of at-risk adults in the context of TSA, enabling targeted risk education and informing future research on the best preventive treatments. To the best of our knowledge, there is no study comparable in cohort size that assesses TSA-associated biomarker risk factors in children. Xu et al., captured in our scoping review, carried out a 2021 study with a cohort size of n = 51 that analyzed the epidemiology of pediatric TSA; however, no risk factor analysis was done in the absence of a control cohort [48]. Miao et al. (2026) did explore biomarkers, finding increased levels of CD26^Low^ cells in pediatric patients with TSA, but it was less clear whether this reflected the TSA phenotype or a broader exacerbation phenotype [49].

The results of this scoping review found that several mixed-cohort papers identified children and younger people as being at higher risk of TSA [8,21,33,38,41] and that this risk may be higher in patients with a pre-existing asthma diagnosis and poor disease control [3,23,29,32,33,36,40,41,43]. Further age-stratified research is crucial to capture the phenotype of the at-risk pediatric TSA patient. Only by identifying the most vulnerable patients will clinicians be able to equip children and caretakers with self-management and safety-netting advice to stay safe in severe weather events.

Implications for Public Health

TSA events are not only a clinical challenge but also a significant public health concern. The sudden surge of asthma presentations can overwhelm emergency services, particularly as many affected patients may not have a prior asthma diagnosis [3]. A recent case-control study (n = 687) by Khademi et al. (2026), captured in our scoping review, found that presentations to emergency services peaked just three to eight hours after storms, thus requiring a rapid response from health services [38]. These factors complicate public health preparedness and resource allocation.

Variability in healthcare-seeking behaviors further complicates system response, as syndromic data report that patients affected by TSA may access care through a range of healthcare services, such as telephone triage lines, primary care services, and EDs [17,28]. This may reflect differences in healthcare systems, access to care, and symptom recognition and perceived severity, especially in pediatric populations where many children may lack a prior asthma diagnosis [13].

The recurrent finding that many pediatric TSA patients have a background of atopic disease suggests potential opportunities for targeted health interventions in high-risk groups, such as patient and parent education and optimization of preventive and rescue therapies. Some studies have explored the use of allergen-specific IgE sensitization to predict at-risk patients, but the clinical practicality of this approach remains uncertain [58]. Further research could clarify whether this approach could be used in combination with regional aeroallergen monitoring.

Strengths

To our knowledge, this is the first scoping review to specifically examine TSA in pediatric populations, addressing an important gap in a body of literature largely focused on adult cohorts. The inclusion of both pediatric and mixed-age studies allowed us to identify findings directly supported by pediatric-specific literature while highlighting the lack of pediatric focus in mixed-cohort papers. The broad temporal coverage (1985-2026) allowed us to capture a broad range of studies from a wide variety of geographical regions. The search of three online databases and blinded screening by two reviewers further strengthened the review process.

Limitations

The age cutoff for pediatrics is debated; while the legal limit of adulthood in many countries is 18 years old, there can be some variability in the pediatric age cutoff in clinical practice. Healthcare services in some countries may continue to treat patients as young people up to the age of 21 years, while in others, they may assess patients as adults from age 14 onward. For the purposes of this review, we assumed the pediatric age cutoffs prescribed by individual studies. This uncertainty highlights the lack of age stratification in studies exploring patient risk factors for TSA and the need to include this in future research.

Next, this scoping review excluded studies not accessible in English. This may have introduced a bias toward TSA research and events occurring in English-speaking regions of the world. Additionally, while this study assessed evidence from three separate electronic databases, gray literature was not included and backward citation searching was not performed.

Finally, many risk factors for TSA are not generalizable to all countries and patient populations. As TSA depends inherently on regional and national climate and allergens, it is important for individual regions to explore which factors put their patients most at risk.

## Conclusions

Exploring pediatric risk factors for TSA has key implications for clinical practice. As climate change is expected to increase the frequency and severity of TSA events, there is an urgent need for age-stratified research to better define the pediatric risk profile. Improved understanding of both clinical and environmental risk factors will be essential to inform targeted prevention strategies, public health preparedness, and clinical management of at-risk children.

To conclude, we synthesized the current evidence on the environmental and clinical factors associated with the development of TSA. We found that factors such as high pollen counts and previous atopic history may be associated with pediatric TSA, but due to the small number of pediatric-specific analyses, definitive conclusions cannot be drawn. Further research is needed to determine the specific environmental and clinical factors that place children at greatest risk.

## Appendices

Appendix A

**Table 3:** Summary of studies excluded for not meaningfully including pediatric age groups. ETSA, epidemic thunderstorm asthma; TSA, thunderstorm asthma

Reference	Overview of study design	Age group captured
Sultana et al. (2019) [59]	Retrospective and hospital-based case-control study of clinical factors for the 2016 Melbourne ETSA epidemic and eight non-ETSA years	15 years or older
Lai et al. (2018) [60]	Retrospective study investigating the relationship between traffic-related air pollution exposure and the 2016 Melbourne ETSA epidemic	15 years or older
Wark et al. (2002) [61]	Case-control study of TSA and non-TSA asthmatics reviewed both acutely and four weeks after ED presentation	16 years or older
Celenza et al. (1996) [62]	Retrospective study of ED records during a June 1994 ETSA event in London to identify environmental factors associated with TSA	16 years or older
Bellomo et al. (1992) [63]	Retrospective case-control (n = 12, n = 16) study of meteorological and clinical data associated with two ETSA events in Melbourne in 1987 and 1989	14 years or older
